# Prevalence of depression and its potential contributing factors in patients with enterostomy: A meta-analytical review

**DOI:** 10.3389/fpsyt.2022.1001232

**Published:** 2022-11-30

**Authors:** Wymann Shao Wen Tang, Li Ling Christine Chiang, Kay Wee Kwang, Melvyn Wei Bin Zhang

**Affiliations:** ^1^Yong Loo Lin School of Medicine, National University of Singapore, Singapore, Singapore; ^2^Lee Kong Chian School of Medicine, Nanyang Technological University Singapore, Singapore, Singapore

**Keywords:** enterostomy, colostomy, ileostomy, depression, mental health

## Abstract

**Objective:**

In patients with intestinal pathologies, the placement of a stoma bag affects multiple aspects of their perceived quality of life. This meta-analysis aims to evaluate the prevalence of depression among patients with enterostomy and to determine the underlying factors that could explain the potential heterogeneity of this prevalence.

**Methods:**

Relevant published studies were identified by searching PubMed, Embase, PsycINFO, Cochrane, CINAHL, Scopus, and Web of Science until May 2022. The random-effects model was used to determine the pooled prevalence of depression among patients with enterostomy using cross-sectional studies from various countries. Meta-regression and subgroup analysis were performed to identify factors contributing to heterogeneity. Quality assessment of the included studies was conducted using the Newcastle-Ottawa scale for nonrandomized studies.

**Results:**

The pooled prevalence of depressive symptoms among patients with enterostomy, as calculated using the random-effects model, was 41.6% (95% confidence interval [CI]: 25.4–59.7%, *Q*-value = 145.794, df = 8, *p* < 0.001, tau^∧^2 = 1.124, I^∧^2 = 94.513). The meta-regression found that mean age and gender were not significant moderators for the observed heterogeneity in prevalence. Subgroup analysis according to the indications for enterostomy formation showed that the prevalence of depression was highest in patients with colorectal cancer, at 34.4% (95% CI: 27.2–42.4%). Subgroup analysis by region showed that patients in Africa had the highest prevalence of depression, at 88.2% (95% CI: 76.1–94.6%), compared to other regions. Subgroup analysis by stoma indication was not significant.

**Conclusion:**

This meta-analysis reports that the pooled prevalence of depression among patients with enterostomy is 41.6%. Indications for enterostomy formation, as well as geographical region, were identified as potential sources of heterogeneity. These findings highlight the need for appropriate psychosocial support and interventions at different stages of enterostomy placement.

## Introduction

Depression affects around 350 million people worldwide; it has been identified by the World Health Organization as the single largest factor contributing to global disability (1). Depression can impair normal functioning, adversely impact patients' quality of life, and increase morbidity and mortality (2).

An intestinal stoma or enterostomy can be defined as a surgically created channel between the intestine and the skin surface. Colostomy and ileostomy are the most common types of enterostomy; the term also comprises cecostomy, jejunostomy, and duodenostomy. The enterostomy can be permanent or temporary; the latter type can be reversed in a subsequent surgical anastomosis. The function of an enterostomy generally includes the diversion of fecal flow as well as the decompression of the distal gut in the context of obstruction. Indications for enterostomy typically include gastrointestinal tract malignancies, inflammatory bowel disease (IBD), and intestinal obstruction. The United Ostomy Associations of America (UOAA) estimates that 725,000 to 1,000,000 patients in the United States live with an ostomy, while 100,000 new patients a year undergo an ostomy-forming surgery (3).

Patients living with an enterostomy can face medical, psychological, and social challenges, which collectively contribute to a reduction in their quality of life (QoL) (4, 5). Medical complications include skin erosions, stomal necrosis, stomal prolapse, parastomal hernias, and dehydration (6, 7). Socially, stoma patients may also be burdened by sexual problems as well as inconveniences with clothing, travel, and worries about the noise and appearances caused by the stoma (8–10). The significant implications of stomas on patients' mental health have also been well reported in the literature. Problems with adjustment have been shown to be common and are affected by multiple factors, such as pre-stoma education, physiological complications, and ability to care for the stoma (8, 11). One study reported that the depression rates in rectal cancer patients with a colostomy were significantly higher than the national norm (12). Emotional responses such as self-disgust and stigma have also been shown to be negatively associated with stoma acceptance (13). In a retrospective cohort analysis of 481 patients with IBD, stoma formation was noted to be independently associated with anxiety and depression. This study also reported an increase in the rates of anxiety and depression among patients who have recently undergone stoma formation (14). Another study found that the psychosocial needs and anxiety arising from the stoma formation were also significant in predicting patients' psychosocial behavioral reactions post-stoma (15).

Despite the presence of multiple studies examining the prevalence of anxiety and depression among these patients, there has not yet been a meta-analysis to establish the prevalence of depressive symptoms in patients with stomas, or to ascertain whether stoma placement is associated with a higher risk of depression in patients (16–24).

Depression has both nonmodifiable risk factors, including family and personal history of depression, as well as modifiable risk factors, such as pain, sleep disturbances, social support, and person–environment fit (25–28). Across cultures, there is wide variability in prevalence estimates of depression, though social demographic correlates and the adverse effects of major depression are consistent (29). In addition, the presence of multimorbidity is a potential risk factor for depression. In one meta-analysis, the risk of depressive disorder was found to be 3 times higher in patients with multimorbidity compared to those without any chronic physical conditions (30). As described above, those with a physical condition like a stoma may suffer from both physical and social dysfunction due to their physical condition, which may contribute to further psychological impairments such as depression (31). Risk factors for depression in patients with stomas have varied widely across studies. They include social isolation, availability of familial support, marital status, socioeconomic status, perceived quality of life and health status, permanence of the stoma, changes in body image, and stoma-related complications, as well as psychiatric and physical comorbidities (18, 20–22, 24, 31).

This meta-analysis therefore aims to establish the pooled prevalence of depression among patients with enterostomy, as well as to assess possible factors associated with the development of depression. This may inform the need to implement relevant interventions to address stoma-related depression.

## Materials and methods

### Literature search strategy

Relevant published studies were identified by searching the following databases (with an end date of May 2022) in order to extract all relevant articles: PubMed, Embase, PsycINFO, Cochrane, CINAHL, Scopus, and Web of Science. The search terms used are summarized as follows: (ostomy or stomas or stoma or enterostomy or cecostomy or colostomy or duodenostomy or ileostomy or jejunostomy or “surgical stoma” or “surgical stomas”) and (depression or “depressive symptoms” or “major depressive disorder” or “depressive illness” or “depressive disorder” or “depressive disease” or “depressive state” or depress^*^ or [(psychological or psychosocial or mental) and (health or illness or state or outcome^*^ or consequence^*^ or need^*^ or wellbeing or well-being)].

### Eligibility criteria and study selection

The inclusion criteria were as follows: (a) the study is a peer-reviewed cross-sectional study; (b) the study involved patients with enterostomies; (c) the study used validated self-reported or clinician-rated tools in defining cases of depression; and (d) the article was in English. Studies that did not report the prevalence of depression among the sampled patients with enterostomies were excluded, as were gray literature and unpublished works.

As this meta-analysis mainly involved extracting data from other published studies, an institutional review board approval was not required.

### Data extraction

Prior to selection, all articles were first de-identified. The title and abstract of each study was then independently reviewed and shortlisted by three authors (CLLC, KKW, and TSWW). Any disagreement was resolved by discussion between the three authors, in consultation with the senior author (MWBZ). The articles were then screened based on their titles and abstracts, and the shortlisted articles were evaluated against the inclusion and exclusion criteria. This selection procedure was conducted in accordance with the Preferred Reporting Items for Systematic Reviews and Meta-analyses (PRISMA) guidelines (32). After the decision on selected articles was finalized, the authors then consolidated the following information in a standard data extraction form: (1) publication details (title; authors); (2) details of the included patients (mean age; population; type of enterostomy; indication of enterostomy); (3) details of the criteria used for defining depression cases (validated instrument used; self-reported or clinician-rated); and (4) percentage of depression cases within the enterostomy population and subpopulations.

### Risk of bias assessment

A quality assessment of the included studies was conducted using the Newcastle-Ottawa scale for nonrandomized studies adapted for cross-sectional studies, as employed in previous studies (33–35). The studies were assessed across the following domains: selection (representativeness of the exposed cohort, satisfactory sample size, response rate and accounting of nonrespondents, and validity of ascertainment of exposure); comparability of outcome groups; and outcomes (assessment of outcome groups and appropriateness of statistical testing).

### Statistical analysis

All statistical analyses were performed using the Comprehensive Meta-Analysis version 3.0 based on the random-effects model and methods established by previous studies (36, 37). Random-effects modeling was used for this analysis because it assumes varying effect sizes between studies due to heterogeneity in study design and population. Heterogeneity between studies was measured using the I^2^ statistic, which describes the percentage of variability among effect estimates beyond that expected by chance. I^2^ values below 25% imply low heterogeneity, I^2^ values of 50% imply moderate heterogeneity, and values of 75% or more imply high heterogeneity.

A meta-regression analysis was performed such that potential factors (both continuous and categorical) that might have contributed to the overall heterogeneity of the pooled effect size could be identified. The regression coefficients and the associated *z*-values and *p*-values are reported in the following sections. Subgroup analysis was undertaken to investigate the effects of categorical variables. We compared the prevalence of depression in enterostomy patients between subgroups based on the country and region of the study, as well as the indication for the enterostomy. Egger's regression test was conducted to determine the presence of publication bias. In cases where significant publication bias was present, the classic fail-safe test was performed to determine the number of missing studies that would be required for the *p*-value of the publication bias among the observed studies to be > 0.05.

## Results

### Literature search results

A total of 6,954 articles were identified from the database search, within which 2,899 duplicate records were identified. All remaining articles [4,055] were screened based on their title and abstract; 3,967 articles were excluded during this step, as they were found to be irrelevant to the aims of the present study and to not be directly related to depression in patients with enterostomy. A total of 88 papers were subsequently selected for full-text assessment. Based on this assessment, 79 studies were excluded for the following reasons: 27 lacked depression-related outcomes; seven had a study population that was not exclusive to enterostomy patients; six were not cross-sectional; eight had no data on depression prevalence in the studied population; nine used instruments that were not validated for defining cases of depression; four studies were not in English; 17 studies full text was unavailable; and 1 study was not yet published in a peer-reviewed journal.

### Characteristics of the included studies

**Figure 1:**
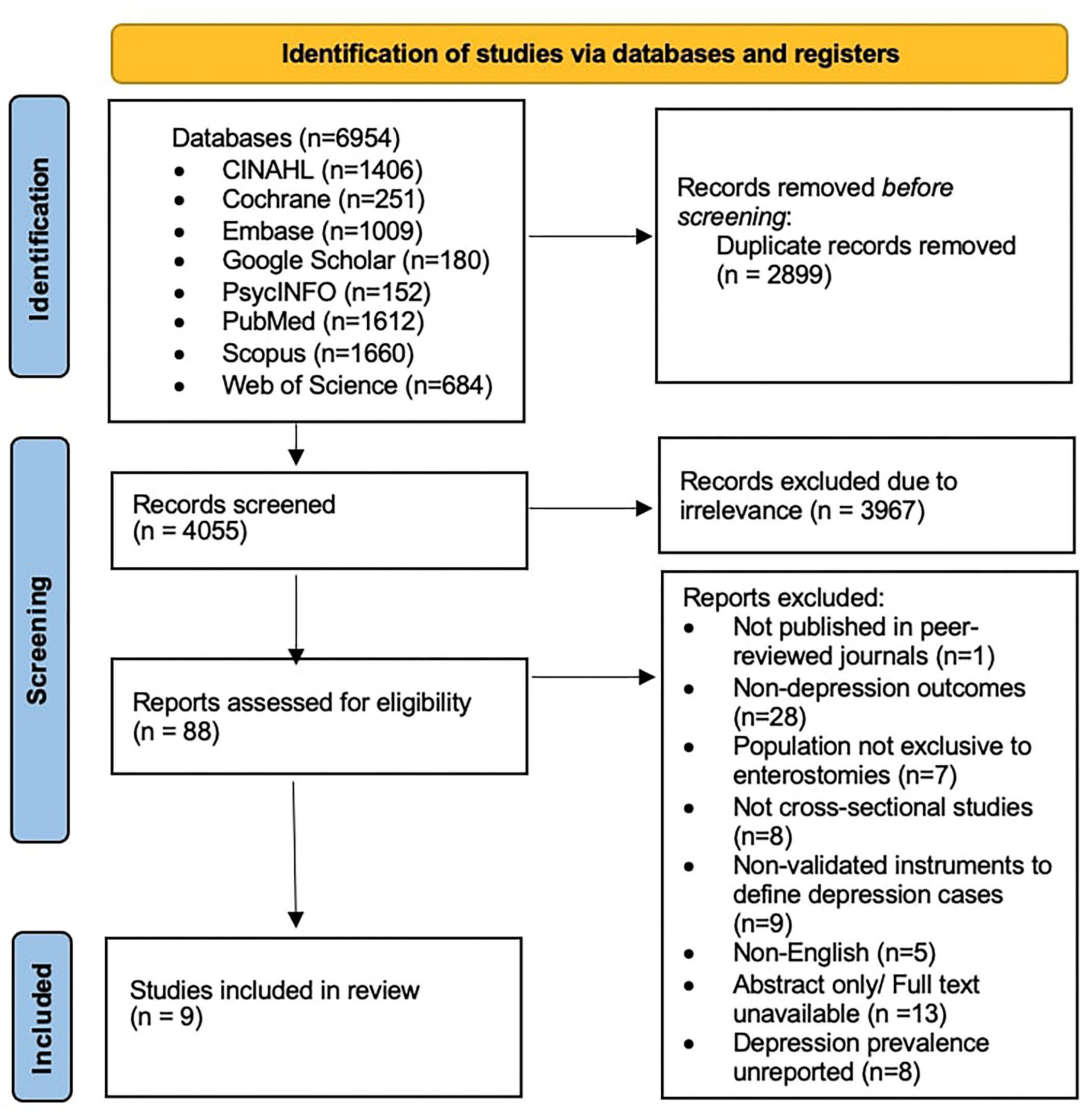
PRISMA flowchart describing the study selection.

The final data set comprises 9 studies with a pooled cohort size of 823 patients. Figure 1 illustrates the selection of the articles, and the characteristics of the included studies are presented in Table 1.

**Table 1:** Characteristics of the included studies.

**References**	**Location**	**Study Design**	**Type of stoma**		**Indications**	**Total sample size (n)**	**Ratio of males to females**	**Mean age (years)**	**Depression assessment method**	**Prevalence of depression (%)**
			**Anatomy**	**Permanent or temporary**						
Al-Aamri et al. (24)	Oman	Cross-sectional	Enterostomy	NA	Inflammatory bowel disease	11	49:51	36.2	Patient Health Questionnaire-9 (PHQ-9) (SR)	12.8
Ananthakrishnan et al. (17)	United States	Cross-sectional	Enterostomy	NA	Inflammatory bowel disease	158	48:52	48–51	Clinician rating	51.9
Jayarajah et al. (18)	Sri Lanka	Cross-sectional	Colostomy, ileostomy	Permanent, temporary	NA	40	68:32	NA	PHQ-9 (SR)	45.0
Moraes et al. (21)	Brazil	Cross-sectional	Colostomy, ileostomy	Permanent, temporary	NA	101	45:55	NA	Beck Depression Inventory (self-reported)	25.7
Norton et al. (16)	United Kingdom	Cross-sectional	Colostomy	Permanent	Fecal incontinence	66	11:58	NA	HADS (SR)	11.0
Rafiei et al. (19)	Iran	Cross-sectional	Colostomy, ileostomy	Permanent, temporary	NA	70	49: 51	62.6	Depression, Anxiety, Stress Scale 21 (SR)	87.1
Rud et al. (23)	Denmark	Cross-sectional	Ileostomy	Permanent, temporary	NA	178	43: 57	58	Major Depression Inventory (Self-reported)	18.0
Song et al. (20)	China	Cross-sectional	Enterostomy	Permanent, temporary	Colorectal cancer	148	72: 28	58.5	HADS (SR); cut-off >/= 9 used)	34.4
Ssewanyana et al. (22)	Uganda	Cross-sectional	Colostomy, ileostomy	Permanent, temporary	NA	51	78: 22	44.04	PHQ-9 (SR)	88.2

NA, not available; SR, self-report; CR, clinician rating.

### Pooled prevalence of depressive symptoms

**Figure 2:**
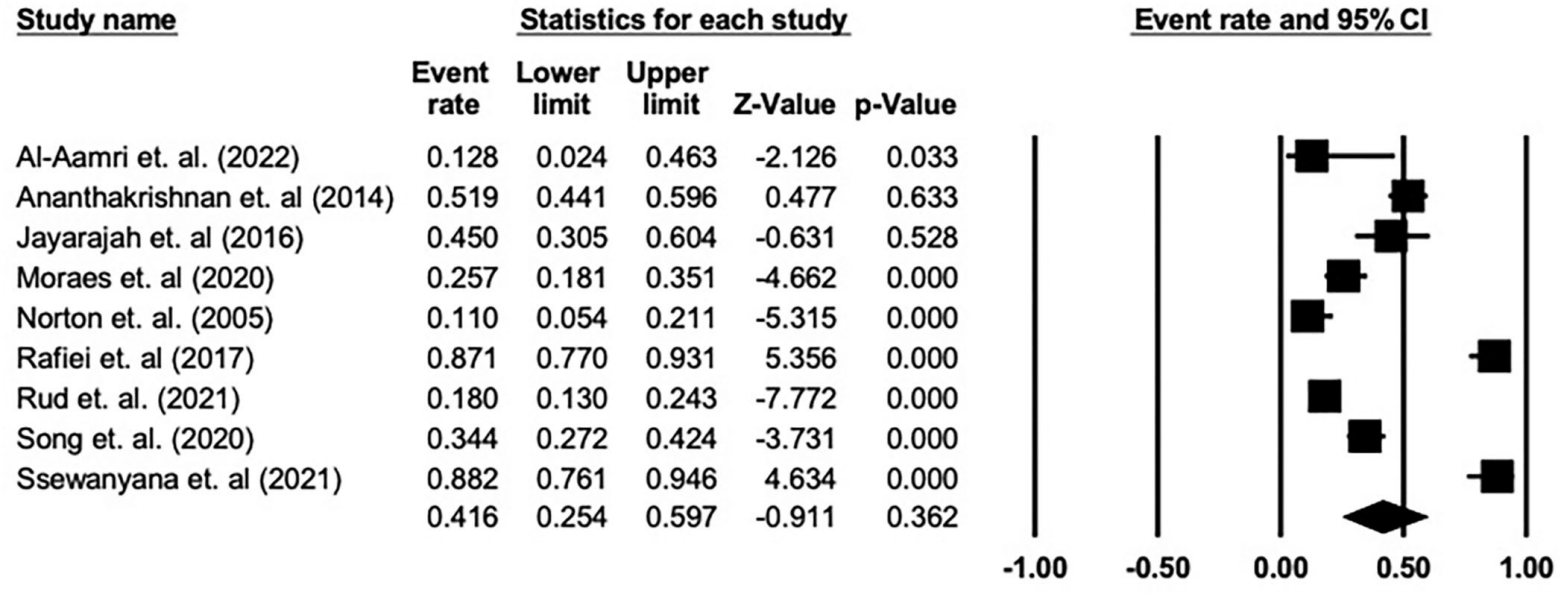
Summary plot for the quality assessment of included studies.

The pooled prevalence of depressive symptoms among patients with enterostomy using the random-effects model was 41.6% [95% confidence interval [CI]: 25.4%−59.7%, *Q*-value = 145.794, df = 8, *p* < 0.001, tau^2^ = 1.124, I^2^ = 94.513]. The meta-analysis shows a statistically significant heterogeneity across the included studies. Figure 2 shows the forest plot generated for these patients and the prevalence of depressive symptoms.

### Factors moderating the prevalence of depressive symptoms

A meta-regression was undertaken to explore the impact of *a priori* sources of heterogeneity across individuals in all studies. It was found that mean age (*B* = −0.082, *Z* = −0.71, *p* = 0.4806) and proportion of male gender (*B* = 3.1063, *Z* = 0.57, *p* = 0.5674) were non-significant moderators (Table 2).

**Table 2:** Meta-regression analysis on the sources of heterogeneity.

**Predictor**	**No. of studies used**	**Univariate coefficient**	***z*** **-value**	***p*** **-value**	**Estimated tau^∧^2**	**R^∧^2**
Mean age of enterostomy patients, years	4	−0.082	−0.71	0.4806	0.1564	0.00
Gender distribution of enterostomy patients, male, %	5	3.1063	0.57	0.5674	0.3353	0.00

Results for

Random-effects meta-regression of the demographic and clinical moderators for patients with enterostomy.

Subgroup effect size analysis of patients with enterostomy.

### Subgroup analysis

A subgroup analysis of the prevalence rates of depression in the context of various categorical moderators was also performed (Table 3). Among all the moderators sampled, the indications for enterostomy, as well as the geographic region of the patients, were significant moderators. With regards to the indications for enterostomy, the pooled prevalence of depression in patients with enterostomy and colorectal cancer was 34.4% (95% CI: 27.2–42.4%). This was the highest prevalence overall; patients with enterostomy and IBD had a depression prevalence of 32.7% (95% CI: 6.7–76.7%) and patients with enterostomy and fecal incontinence had a prevalence of 11.0% (95% CI: 5.4–21.1%), *p* = 0.003.

**Table 3:** Subgroup analysis on selected sources of heterogeneity.

**Subgroup**	**No. of studies**	**Pooled prevalence (%)**	**95% CI (%)**	***p*** **-value in between-group comparison**
Indication for stoma: Colorectal cancer	1	34.4	27.2–42.4	0.003
Indication for stoma: Inflammatory bowel disease	2	32.7	6.7–76.7	
Indication for stoma: Fecal incontinence	1	11.0	5.4–21.1	
Overall	4	29.4	23.5–36.1	
Region: Europe (1)	2	15.4	9.7–23.6	0.000
Region: Asia (2)	2	37.7	28.7–47.6	
Region: Middle East (3)	2	52.1	2.5–97.9	
Region: North America (4)	1	51.9	44.1–59.6	
Region: South America (5)	1	25.7	18.1–35.1	
Region: Africa (6)	1	63.0	8.9–96.7	
Overall	9	36.7	32.2–41.5	

In terms of geographic region, the pooled prevalence of depression in patients with enterostomy in Africa was the highest, at 88.2% (95% CI: 76.1–94.6%). The prevalences in the Middle East and North America were the next highest, at 52.1% (95% CI: 2.5–97.9%) and 51.9% (95% CI: 44.1–59.6%), respectively. The prevalence of depression was lowest in Europe, at 15.4% (95% CI: 9.7–23.6%).

### Results of the risk of bias assessment and publication bias

**Figure 3:**
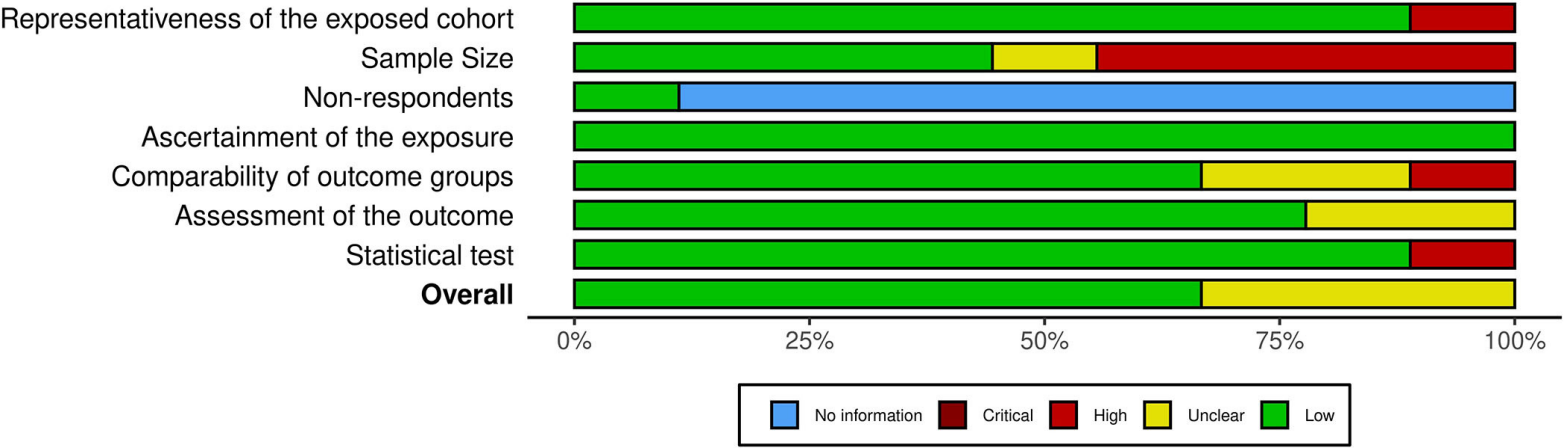
Forest plot showing the prevalence of depression among patients with enterostomy in the included studies.

A quality assessment of the included studies was performed using the Newcastle-Ottawa scale for nonrandomized studies adapted for cross-sectional studies; the summary of this assessment is presented in Figure 3. Further details on the assessment are presented in Supplementary Table 3. All of the included studies were deemed to be at least satisfactory.

Publication bias was also tested for using funnel plots and Egger's regression test. Bias was evident in the meta-analysis of all studies (intercept = 1.46, 95% CI: −7.30–10.22, *t* = 0.40, df = 7, *p* = 0.704). Based on the classic fail-safe test, 70 additional studies would required for every study included in this meta-analysis in order to nullify these bias results.

## Discussion

This study represents, to the best of our knowledge, the first meta-analysis that examines the prevalence of depression among patients with enterostomy across different countries. The aggregated prevalence of depression in patients with enterostomy was 41.6%, suggesting that approximately 4 in 10 of these patients are likely to be clinically depressed. This aggregated prevalence was around 10 times higher than the estimated global prevalence of depression of 3.8% (38), suggesting that the presence of an enterostomy is strongly associated with depression. This is not unexpected, as other studies have demonstrated a clear relationship between stomas and body image issues, feelings of self-disgust, and a reduction in perceived quality of life due to various inconveniences and complications arising from the stoma (4, 5, 13).

Meta-regression analysis revealed that the proportion of male gender and the mean age of the patients were not significant moderators and did not account for the high heterogeneity in the pooled prevalence of depression reported. It is interesting to note that gender was not a significant moderator, given the known gender difference in the prevalence of depression between women and men (2:1) (39). However, our findings vary from the outcomes of previous studies where mixed outcomes have been demonstrated for the effects of gender on the psychosocial wellbeing of patients with enterostomy. A study by Gautam et al. on the effect of gender on psychosocial adjustment in Nepalese patients with colorectal cancer and ostomies found that men had significantly lower psychosocial adjustment scores and reported more negative emotions (40). In contrast, Krouse et al. reported that female survivors of rectal cancer with an ostomy had significantly worse psychological wellbeing (41). These differences were attributed to differences in acceptance and social engagement for men, and to worries relating to isolation and familial distress for women. It should, however, be noted that these studies used quality-of-life tools to measure psychosocial wellbeing and were not directly measuring the prevalence of clinical depression, and were therefore not included in this meta-analysis.

Our findings on the effect of age on depression prevalence may also differ from individual studies not included in this meta-analysis, such as the study by Park et al. (31) that found that older patients with stomas have a higher prevalence of depression, at 50.7%. This finding was associated with factors such as social isolation, poor financial status, and perceived health status (31). With their increased likelihood of disability and of having multiple chronic medical conditions, the elderly population is, as a group, vulnerable to depression; this may be further exacerbated by the presence of a stoma, as well as by the underlying condition requiring stoma formation (42). It is also noteworthy that the presence of a stoma can significantly worsen the symptoms that are more prominent in elderly depression, such as insomnia and somatic complaints, due to the discomfort and inconvenience arising from the stoma (43).

The subgroup analysis showed that aggregated depression prevalence varied as a function of the underlying indication for an enterostomy. The pooled prevalence of depression was highest in patients with colorectal cancer, followed by patients with inflammatory bowel disease and fecal incontinence. This finding corroborates previous studies that have established an increased prevalence of depression compared to the general population in both patients with colorectal cancer (ranging from 1.6 to 57%) and patients with inflammatory bowel disease (22–28.5%) (44, 45). The circumstances affecting this difference are likely to be multifactorial in origin and could possibly be attributed to the prognosis of the underlying condition, as well as to extra-stoma symptoms related to it. This correlates with Orive et al.'s cohort study on patients with colorectal cancer, which demonstrated that on follow-up, worsening depression was associated with having more comorbidities, having a stoma, and experiencing complications after interventions (46). Hu et al. in constructing a risk nomogram for postoperative depression in patients with colorectal cancer, revealed that comorbidities, postoperative complications, and the presence of a stoma were significant indicators for depression, among other factors including socio-economic status, gender, and functional status (47). In patients with colorectal cancer, the development of depression may be linked to a poor prognosis associated with the diagnosis; other contributing factors may include physical stress such as pain, fatigue, and changes in stool frequency arising from the disease and its treatments, such as surgery and chemotherapy (48, 49).

IBD has also been strongly correlated with depression in established studies included in previous meta-analyses by Barberio et al. and Neuendorf et al. whereby the pooled prevalence of depression was higher in female patients, patients with Crohn's disease, and patients with more active disease (45, 50). Interestingly, Luo et al. have also corroborated a possible bidirectional relationship between IBD and depression (51). It has also been posited that pro-inflammatory mediators associated with IBD can also be a contributing factor to depression (52, 53). In IBD, other contributing factors include uncertainty of the underlying prognosis as well as cancer risk (53). Treatment itself can also add to the risk of the development of depression—especially so with the use of pharmacological interventions such as corticosteroids and antibiotics, which include depression as a potential side effect (54, 55). While our study depicted that the differences in depression between patients with different indications for an enterostomy may also be due to differences in the duration of time living with a stoma, we were unable to conduct further meta-regression analysis for the duration of time living with stoma, or further subgroup analysis on the effects of the permanence of the stoma, in this meta-analysis.

In terms of geographic region, the pooled prevalence of depression in enterostomy patients was highest in African nations, followed by the Middle East, North America, Asia, South America, and Europe. Based on the findings of the 2019 Global Burden of Disease Study on the global and regional burden of mental disorders, the prevalence of depressive disorders across regions correlated well with the differences in depression rates in patients with stoma seen in our results (56). The prevalence of depressive disorders in the general population was also found to be highest in sub-Saharan Africa (4,540.1 per 100,000 people) followed by the Middle East (4,348.9 per 100,000 people) and by North America (4,270.3 per 100,000 people) (56). Our study included 2 papers from Asian countries, i.e., Sri Lanka and China. The prevalence of depressive disorders in the general population is higher in South Asia (3,794.7 per 100,000 people) compared to East Asia (2,720.1 per 100,000 people) (56). The higher rates of depression in South Asia could have contributed to the overall higher prevalence in Asia of depression in people with stoma. The differences in the overall prevalence of depressive disorders across countries could be one factor accounting for the differences in prevalence rates of depression in patients with stoma.

Other than the overall difference in prevalence rates across cultures, specific factors accounting for regional differences in depression rates among patients with enterostomy could include differences in standards of psychosocial support, the availability of facilities and interventions aimed at providing support for people with stoma, and cultural factors such as social stigma and awareness about living with a stoma. This is a significant consideration, as it has been shown that the presence of, and post-surgery access to, a psychosocial intervention program is beneficial to the mental health and quality of life of people who recently underwent stoma formation. A meta-analysis found that psychosocial interventions during the post-intervention period had a significant benefit on quality of life in patients with colorectal cancer (57). Similarly, a study found that a psychosocial intervention program for patients with colorectal cancer and stoma found significant effects on acceptance of the stoma in the intervention group (58). However, the exact factors that account for the differences in depression rates in patients across different regions, such as post-surgery care and support, are difficult to generalize, and further research is being conducted to explore this discrepancy.

Upcoming studies, such as the mixed-method Stoma Care For Improvement Research (STARFISH) study, can shed more light on the differences in stoma care between high-income and low- and middle-income countries (59). This will include exploring the amount of support received, the challenges faced, and access to services and supplies for the care of stomas in low- and middle-income countries (59). Further research could explore the differences across cultures that could influence depression rates and psychological distress in patients with stoma.

### Strengths

The strengths of this review include the comprehensiveness of the search strategy, which covered a wide range of databases and studies across different regions and countries, as well as the inclusion of meta-regression and subgroup analysis. Our results should therefore be relevant to clinicians and multidisciplinary teams in terms of raising awareness of the prevalence of depression among patients with enterostomy, and in terms of screening and preventive strategies to reduce the detrimental effects of depression on the care of these patients.

### Limitations

However, due to the limited availability of studies with appropriate datasets, we were unable to perform further detailed subgroup analyses, such as comparing clinician-rated and self-reported depression scales, or meta-regression analysis on the mean duration of enterostomy. Furthermore, due to the nature of the data reported (e.g., reporting of odds ratios instead of prevalence), some studies could not be included; their inclusion would have improved the strength of our subgroup analysis in domains such as the mean age of patients with stomas. Furthermore, since all of the studies included were cross-sectional, causality or temporal association between depression and having an enterostomy cannot be firmly established. Lastly, despite the meta-regression analysis conducted, the effect of gender on depressive symptoms and prevalence in patients with enterostomies may not yet be clearly established, in view of variable findings from other studies using different outcome measures.

### Clinical relevance and implications

These findings affirm the need for continuous psychosocial support for people with stoma. This suggests that there is room for additional roles to be filled by various health care disciplines in caring for patients with enterostomy, such as through pre- and post–stoma formation interventions. For instance, Koç et al. have shown that the implementation of pre-stoma “prehabilitation,” including a preoperative “introduction” of the stoma appliance to the patient and postoperative education, has led to improved self-care ability and quality of life, as well as reduced propensity to anxiety and depression (60). A greater understanding of the factors influencing the development of depression in patients with enterostomy will also be helpful in efforts to develop appropriate interventions to reduce the morbidity caused by depression. Areas of interest include factors such as pre-surgery depression, self-efficacy, illness perception, and coping mechanisms, as identified by Foster et al. (61) and Knowles et al. (62).

## Conclusion

This meta-analysis reports that the pooled prevalence of depression among patients with enterostomy is 41.6%. Upon meta-regression analysis, age and gender were not found to explain the high heterogeneity in the pooled prevalence, while the indications for enterostomy formation and geographic region were identified as potential sources of heterogeneity. This meta-analysis also draws attention to the importance of assessing the psychological aspects of health in surgical patients, including patients with enterostomy, and discusses possible factors influencing the development of clinical depression. Given the high prevalence of depression, it is important for further studies to elucidate psychopathological pathways in the development of clinical depression among patients with enterostomy, and for measures to be developed to provide appropriate psychosocial support and interventions at different stages of the enterostomy creation process.

## Data availability statement

The original contributions presented in the study are included in the article/Supplementary material, further inquiries can be directed to the corresponding author.

## Author contributions

All authors listed have made a substantial, direct, and intellectual contribution to the work and approved it for publication.

## Conflict of interest

The authors declare that the research was conducted in the absence of any commercial or financial relationships that could be construed as a potential conflict of interest.

## Publisher's note

All claims expressed in this article are solely those of the authors and do not necessarily represent those of their affiliated organizations, or those of the publisher, the editors and the reviewers. Any product that may be evaluated in this article, or claim that may be made by its manufacturer, is not guaranteed or endorsed by the publisher.
