# Supplementary material for: Prevalence of depression and its potential contributing factors in patients with enterostomy: A meta-analytical review
Source: Front Psychiatry. 2022 Nov 30;13:1001232. doi: 10.3389/fpsyt.2022.1001232 (PMC9756805; doi:10.3389/fpsyt.2022.1001232)

**Supplementary Materials**

Supplementary Table 1: Description of results from included studies

| Study (year) | Permanent stoma | | Temporary Stoma | | Ileostomy | | Colostomy | |
| --- | --- | --- | --- | --- | --- | --- | --- | --- |
|  | No. (% of Total) | Prevalence of depression (%) | No. (% of Total) | Prevalence of depression (%) | No. (% of Total) | Prevalence of depression (%) | No. (% of Total) | Prevalence of depression (%) |
| Al-Aamri et al., 2022 | NA | NA | NA | NA | NA | NA | NA | NA |
| Ananthakrishnan et al., 2014 | NA | NA | NA | NA | NA | NA | NA | NA |
| Jayarajah et al., 2016 | NA | NA | NA | NA | 11 (27.5) | NA | 29 (72.5) | NA |
| Moraes et al., 2020 | NA | NA | NA | NA | 6 (5.94) | 50 | 95 (94.06) | 24.2 |
| Norton et al., 2005 | NA | NA | NA | NA | NA | NA | 66 (100) | 11.0 |
| Rafiei et al., 2017 | NA | NA | NA | NA | 3 (4.29) | NA | 67 (95.71) | NA |
| Rud et al., 2021 | NA | NA | NA | NA | 178 (100) | NA | NA | NA |
| Song et al., 2020 | 44 (29.73) | 47.7 | 104 (70.27) | 28.8 |  | NA | NA | NA |
| Ssewanyana et al., 2021 | 6 (11.76) | 66.6 | 45 (88.24) | 91.1 | 12 (23.53) | 91.7 | 39 (76.47) | 87.2 |

Supplementary Table 2: Search Strategy for databases searched

| Pubmed | (ostomy OR ostomate* OR stomas OR stoma OR enterostomy OR cecostomy OR colostomy OR duodenostomy OR ileostomy OR jejunostomy OR “surgical stoma” OR “surgical stomas” OR "Surgical Stomas"[Mesh] OR "Enterostomy"[Mesh]) AND (Depression OR “depressive symptoms” OR “major depressive disorder” OR “depressive illness” OR “depressive disorder” OR “depressive disease” OR “depressive state” OR depress* OR ((Psychological OR psychosocial OR mental) AND (health OR illness OR state OR outcome* OR consequence* OR need* OR wellbeing OR well-being)) OR "Depressive Disorder"[Mesh] OR "Depression"[Mesh] OR "Mental Health"[Mesh]) |
| --- | --- |
| Cochrane | (ostomy OR stomas OR stoma OR enterostomy OR cecostomy OR colostomy OR duodenostomy OR ileostomy OR jejunostomy OR “surgical stoma” OR “surgical stomas”) OR (Ostomy MeSH AND Depression OR “depressive symptoms” OR “major depressive disorder” OR “depressive illness” OR “depressive disorder” OR “depressive disease” OR “depressive state” OR depress* OR (Psychological OR psychosocial OR mental) AND (health OR illness OR state OR outcome* OR consequence* OR need* OR wellbeing OR well-being)) |
| CINAHL | (ostomy OR stomas OR stoma OR enterostomy OR cecostomy OR colostomy OR duodenostomy OR ileostomy OR jejunostomy OR “surgical stoma” OR “surgical stomas”) AND (Depression OR “depressive symptoms” OR “major depressive disorder” OR “depressive illness” OR “depressive disorder” OR “depressive disease” OR “depressive state” OR depress* OR (Psychological OR psychosocial OR mental) AND (health OR illness OR state OR outcome* OR consequence* OR need* OR wellbeing OR well-being)) |
| PsychINFO | (exp colostomy/ OR (ostomy or stomas or stoma or enterostomy or cecostomy or colostomy or duodenostomy or ileostomy or jejunostomy or surgical stoma or surgical stomas).ab,ti.) AND (exp major depression/ or atypical depression/ or "depression (emotion)"/ or seasonal affective disorder/  exp affective disorders/ or exp Mental Health/ OR (Depression or depressive symptoms or major depressive disorder or depressive illness or depressive disorder or depressive disease or depressive state or depress*).ab,ti. OR (Psychological or psychosocial or mental).mp. and (health or illness or state or outcome* or consequence* or need* or wellbeing or well-being).ab,ti. [mp=title, abstract, heading word, table of contents, key concepts, original title, tests & measures, mesh word] |
| Google Scholar | (ostomy OR ostomate OR ostomates OR stomas OR stoma OR enterostomy OR surgical stoma OR surgical stomas) AND (intitle:depression OR intitle:psychological OR intitle:mental OR intitle:psychosocial OR intitle:depressive) |
| Scopus | TITLE-ABS-KEY(ostomy OR stomas OR stoma OR enterostomy OR cecostomy OR colostomy OR duodenostomy OR ileostomy OR jejunostomy OR “surgical stoma” OR “surgical stomas”) AND TITLE-ABS-KEY(Depression OR “depressive symptoms” OR “major depressive disorder” OR “depressive illness” OR “depressive disorder” OR “depressive disease” OR “depressive state” OR depress* OR (Psychological OR psychosocial OR mental) AND (health OR illness OR state OR outcome* OR consequence* OR need* OR wellbeing OR well-being)) |
| Embase | ('stoma'/exp OR 'enterostomy'/exp OR ostomy:ti,ab,kw OR stomas:ti,ab,kw OR stoma:ti,ab,kw OR enterostomy:ti,ab,kw OR cecostomy:ti,ab,kw OR colostomy:ti,ab,kw OR duodenostomy:ti,ab,kw OR ileostomy:ti,ab,kw OR  jejunostomy:ti,ab,kw OR 'surgical stoma':ti,ab,kw OR 'surgical stomas':ti,ab,kw) AND ('depression'/exp OR 'major affective disorder'/exp OR 'mental health'/exp OR depression:ti,ab,kw OR 'depressive symptoms':ti,ab,kw OR 'major depressive disorder':ti,ab,kw OR 'depressive illness':ti,ab,kw OR 'depressive disorder':ti,ab,kw OR 'depressive disease':ti,ab,kw OR 'depressive state':ti,ab,kw OR depress*:ti,ab,kw (psychological:ti,ab,kw OR psychosocial:ti,ab,kw OR mental:ti,ab,kw) AND (health:ti,ab,kw OR  illness:ti,ab,kw OR state:ti,ab,kw OR outcome*:ti,ab,kw OR consequence*:ti,ab,kw OR need*:ti,ab,kw OR wellbeing:ti,ab,kw OR 'well being':ti,ab,kw)) |
| Web of Science | **(**(TI=(ostomy OR stomas OR stoma OR enterostomy OR cecostomy OR colostomy OR duodenostomy OR ileostomy OR jejunostomy OR “surgical stoma” OR “surgical stomas”)) OR AB=(ostomy OR stomas OR stoma OR enterostomy OR cecostomy OR colostomy OR duodenostomy OR ileostomy OR jejunostomy OR “surgical stoma” OR “surgical stomas”)) OR AK=(ostomy OR stomas OR stoma OR enterostomy OR cecostomy OR colostomy OR duodenostomy OR ileostomy OR jejunostomy OR “surgical stoma” OR “surgical stomas”) AND ((TI=(Depression OR “depressive symptoms” OR “major depressive disorder” OR “depressive illness” OR “depressive disorder” OR “depressive disease” OR “depressive state” OR depress*)) OR AB=(Depression OR “depressive symptoms” OR “major depressive disorder” OR “depressive illness” OR “depressive disorder” OR “depressive disease” OR “depressive state” OR depress*)) OR AK=(Depression OR “depressive symptoms” OR “major depressive disorder” OR “depressive illness” OR “depressive disorder” OR “depressive disease” OR “depressive state” OR depress*) ((TI=((Psychological OR psychosocial OR mental) AND (health OR illness OR state OR outcome* OR consequence* OR need* OR wellbeing OR well-being))) OR AB=((Psychological OR psychosocial OR mental) AND (health OR illness OR state OR outcome* OR consequence* OR need* OR wellbeing OR well-being))) OR AK=((Psychological OR psychosocial OR mental) AND (health OR illness OR state OR outcome* OR consequence* OR need* OR wellbeing OR well-being)) |

Supplementary Table 3: Quality assessment of included articles using the Newcastle-Ottawa scale adapted for cross-sectional studies

| Study | Selection | | | | Comparability | Outcome | | Overall score |
| --- | --- | --- | --- | --- | --- | --- | --- | --- |
|  | Representativeness of the exposed cohort | Sample Size | Non-respondents | Ascertainment of the exposure | Comparability of outcome groups | Assessment of the outcome | Statistical test |  |
| Al-Aamri et al, 2022 | * | * |  | ** | ** | * | * | 8 |
| Ananthakrishnan et al, 2014 | * | * | * | ** | ** | ** | * | 10 |
| Jayarajah et al, 2016 | * |  |  | ** | * | * | * | 6 |
| Moraes et al, 2020 | * | * |  | ** | ** | * | * | 8 |
| Norton et al, 2005 | * |  |  | ** | * | * |  | 5 |
| Rafiei et al, 2017 | * |  |  | ** | ** | * | * | 7 |
| Rud et al, 2021 | * |  |  | ** | ** | * | * | 7 |
| Song et al, 2020 | * | * |  | ** | ** | * | * | 8 |
| Ssewanyana et al, 2021 |  |  |  | ** | ** | * | * | 6 |

Supplementary Figure 1- Funnel plot for detecting publication bias


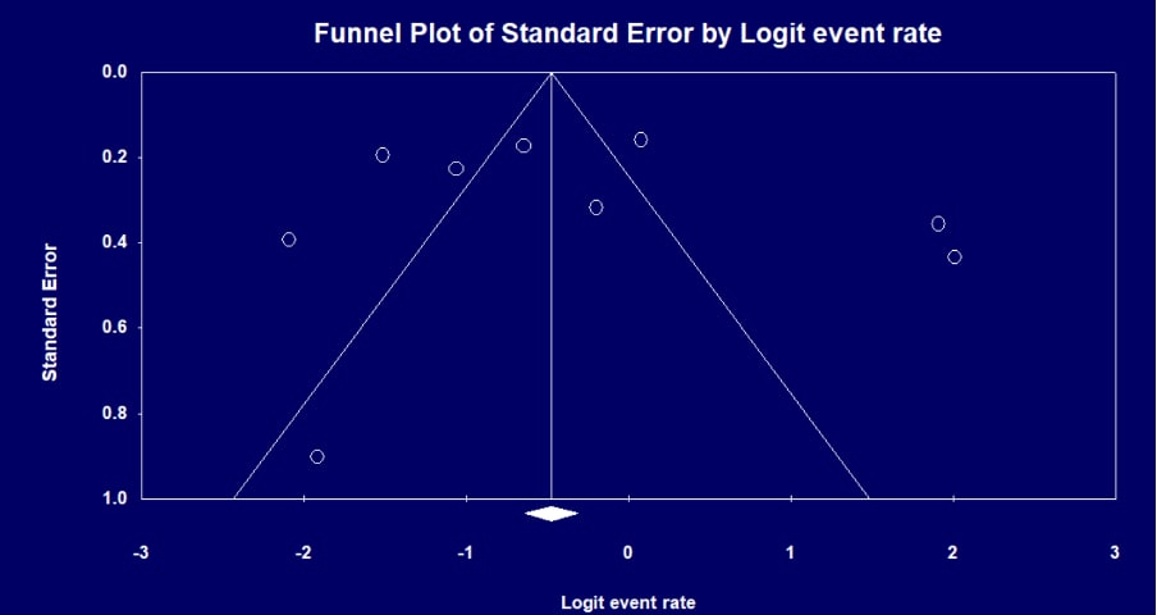

Supplement: Supplementary file 1 [file Data_Sheet_1.docx]
